# The Human Glycome Atlas project for cataloging all glycan-related omics data in human

**DOI:** 10.1093/glycob/cwae052

**Published:** 2024-07-26

**Authors:** Kiyoko F Aoki-Kinoshita, Yukie Akune-Taylor, Hiromune Ando, Kiyohiko Angata, Morihisa Fujita, Jun-ichi Furukawa, Hiroyuki Kaji, Koichi Kato, Ken Kitajima, Yasuhiko Kizuka, Yusuke Matsui, Kazuki Nakajima, Shoko Nishihara, Tetsuya Okajima, Kazuma Sakamoto, Chihiro Sato, Morten Thaysen-Andersen, Akira Togayachi, Hirokazu Yagi, Achille Zappa, Kenji Kadomatsu

**Affiliations:** GaLSIC, Soka University, 1-236 Tangi-machi, Hachioji, Tokyo, 192-8577, Japan; iGCORE, Nagoya University, Furo-cho, Chikusa-ku, Nagoya, 464-8601, Japan; GaLSIC, Soka University, 1-236 Tangi-machi, Hachioji, Tokyo, 192-8577, Japan; iGCORE, Gifu University, 1-1, Yanagido, Gifu, 501-1193, Japan; GaLSIC, Soka University, 1-236 Tangi-machi, Hachioji, Tokyo, 192-8577, Japan; iGCORE, Gifu University, 1-1, Yanagido, Gifu, 501-1193, Japan; iGCORE, Nagoya University, Furo-cho, Chikusa-ku, Nagoya, 464-8601, Japan; iGCORE, Nagoya University, Furo-cho, Chikusa-ku, Nagoya, 464-8601, Japan; ExCELLS, National Institutes of Natural Sciences, Higashiyama-5-1, Myodaijicho, Okazaki, Aichi, 444-0864, Japan; iGCORE, Nagoya University, Furo-cho, Chikusa-ku, Nagoya, 464-8601, Japan; iGCORE, Gifu University, 1-1, Yanagido, Gifu, 501-1193, Japan; iGCORE, Nagoya University, Furo-cho, Chikusa-ku, Nagoya, 464-8601, Japan; iGCORE, Gifu University, 1-1, Yanagido, Gifu, 501-1193, Japan; GaLSIC, Soka University, 1-236 Tangi-machi, Hachioji, Tokyo, 192-8577, Japan; Graduate School of Medicine, Nagoya University, 64 Tsurumai-cho, Showa-ku, Nagoya, 466-8550, Japan; Graduate School of Medicine, Nagoya University, 64 Tsurumai-cho, Showa-ku, Nagoya, 466-8550, Japan; iGCORE, Nagoya University, Furo-cho, Chikusa-ku, Nagoya, 464-8601, Japan; iGCORE, Nagoya University, Furo-cho, Chikusa-ku, Nagoya, 464-8601, Japan; Macquarie University, 13 Research Park Dr, Macquarie Park NSW 2109, Australia; GaLSIC, Soka University, 1-236 Tangi-machi, Hachioji, Tokyo, 192-8577, Japan; ExCELLS, National Institutes of Natural Sciences, Higashiyama-5-1, Myodaijicho, Okazaki, Aichi, 444-0864, Japan; GaLSIC, Soka University, 1-236 Tangi-machi, Hachioji, Tokyo, 192-8577, Japan; iGCORE, Nagoya University, Furo-cho, Chikusa-ku, Nagoya, 464-8601, Japan

**Keywords:** bioinformatics, database, glycogenes, glycoproteomics, human glycome, knowledgebase

## Abstract

The Human Glycome Atlas (HGA) Project was launched in April 2023, spearheaded by three Japanese institutes: the Tokai National Higher Education and Research System, the National Institutes of Natural Sciences, and Soka University. This was the first time that a field in the life sciences was adopted by the Ministry of Education, Culture, Sports, Science and Technology (MEXT) for a Large-scale Academic Frontiers Promotion Project. This project aims to construct a knowledgebase of human glycans and glycoproteins as a standard for the human glycome. A high-throughput pipeline for comprehensively analyzing 20,000 blood samples in its first five years is planned, at which time an access-controlled version of a human glycomics knowledgebase, called TOHSA, will be released. By the end of the final tenth year, TOHSA will provide a central resource linking human glycan data with other omics data including disease-related information.

## Introduction

In April 2023, the Human Glycome Atlas (HGA) Project was initiated (Kadomatsu 2024), marking a significant milestone in the realm of life sciences. Led by three prominent Japanese institutions—the Institute for Glyco-core Research (iGCORE) at Nagoya University and Gifu University, the Exploratory Research Center on Life and Living Systems (ExCELLS) within the National Institutes of Natural Sciences, and the Glycan and Life Systems Integration Center (GaLSIC) at Soka University—this pioneering endeavor is the first large-scale academic Frontiers promotion project supported by the Ministry of Education, Culture, Sports, Science and Technology (MEXT) of Japan in the life sciences field. The primary objective of the project is to establish a comprehensive knowledgebase of human glycans and glycoconjugates as well as glycan-related genes and diseases, setting a standard for the human glycome. Over the course of its initial five years, the project plans to implement a high-throughput pipeline aimed at analyzing 20,000 blood samples. Upon completion of this phase, an access-controlled version of a knowledgebase called TOHSA will be released. By its culmination in the tenth year, TOHSA is envisioned to evolve into a central data resource, seamlessly integrating human glycan data with other omics datasets, thereby facilitating a deeper understanding of disease-related mechanisms involving glycans. Upon completion of this project, an open version of TOHSA, without access restrictions, will also be made available for true FAIRness.

While this project has just begun, much progress has been made in the first year and a half, with the establishment of standard operating procedures (SOPs) for glycoproteomics, glycomics, cohort sample processing, etc. These data are being collated into a standardized repository developed in-house to ensure that all metadata is stored appropriately. FAIR principles (Wilkinson et al. 2016) will be upheld throughout, especially when the access-controlled TOHSA knowledgebase is made available. At that time, it is expected that the SOPs will also be made available such that further collaborations with international institutes can be established.

Through this HGA project, we aim to provide the foundation for the field to take the next step in life sciences following the Human Genome and Proteome Projects. By providing information in a FAIR manner, the data in TOHSA will be easily integrated with other omics data, including lipidomics, metabolomics and proteomics. As glycosylation often has been overlooked in medicine, this integration should help propel research in the medical and life sciences forward greatly, especially with advances in artificial intelligence. Ultimately, this project will be able to propose a reference human glycome, combined with other information crucial to understand human diversity and disease.

The research plan for HGA consists of four segments:

Segment 1: Creation of a detailed glycopeptide list (called a reference map) of all human glycoproteins. This will be produced by preemptively performing deep glycoproteomics coupled with glycopeptide fractionation. In addition to the common MS^2^-based identification (Kaji 2021a), this data acquisition will include the results of a high depth identification using the established IGOT-LC/MS method (Kaji 2021b) in combination with MS1-based glycopeptide deductions (Hiono et al. 2024). This map enables a much larger number of glycopeptides to be identified in a single LC/MS analysis than is possible with MS^2^, and it can fully support the cohort studies in Segment 2.

Segment 2: Creation of a human glycan catalog. The human glycoprotein reference map will be utilized to perform glycoproteome characterization of large populations. In addition, through a “Total Glycomics” approach (Yoshida et al. 2016; Furukawa et al. 2021), glycan structures of five categories of *N*-linked glycans, *O*-linked glycans, glycosphingolipid (GSL)-glycans, glycosaminoglycans, and free oligosaccharides can be simultaneously and quantitatively obtained for each individual. Furthermore, we have established sialic acid linkage-specific alkylamidation (SALSA) to distinguish sialylated glycan isomers by MS analysis (Hanamatsu et al. 2018) and the SALSA method has been introduced into *N*-glycans, GSL-glycans, and O-glycan analysis. To obtain these glycan structure data, collaborations will be necessary with many biobanks. These individual glycan structures and proteomic data will then be catalogued together with relevant clinical epidemiological information in a highly secure Cohort sample database developed internally. Since glycans vary between individuals and even within the same individual across tissues and conditions, this information will allow the identification of the characteristics of glycans specific to disease, race, age, gender, etc.

Segment 3: Construction of a glycan biosynthesis atlas. Approximately 200 kinds of glycosyltransferases are involved in the biosynthesis of human glycans. Comprehensive information on the gene expression, subcellular distribution, and enzymatic properties (activity and substrate specificity) of these enzymes will be obtained to create a highly accurate glycan biosynthesis simulator. Since most of these enzymes are localized within the Golgi apparatus, the molecular network that defines their location within the Golgi will be highlighted based on bioimaging and omics analyses (Yagi et al. 2024). These findings will provide the basis for the creation of artificial cells with controlled glycosylation.

Segment 4: The data obtained in Segments 1–3 will be stored together with clinical and phenotypic data in the knowledgebase TOHSA, and the open access version will have user and application programming interfaces that can be easily accessed by all researchers and shared worldwide. Initially, around year five of this project, due to the nature of the samples, TOHSA will be only made available in an access-controlled manner, after ethical and confidentiality agreements have been made. At this point, we expect to have analyzed and catalogued approximately 10,000 blood samples from a variety of patient cohorts, including Alzheimer’s, dementia and older aged patients. Near the end of this project, an open-access version of TOHSA will also be made available to the public. During this latter half, this project also aims to analyze other organs and tissues.

Segments 1–4 will be executed within Research Goal 1 of the HGA project, “Establishment of Glycan Information Infrastructure”. Moreover, there are two other Research Goals under this project, namely, “Establishment of Equipment and Technology Infrastructure” as Research Goal 2 to support these four segments, and “Establishment of Collaborative Infrastructure” as Research Goal 3. This final research goal is of most importance as it will be the foundation for collaborations at the international level. We hope that the standardized protocols established in this project will be easily distributable and applicable to other cohort samples such that a trustworthy and truly worldwide resource for human glycomics can be established. As this information will also be integrated with other omics information including genomics, metabolomics, proteomics, and others, this will have far-reaching impacts and lasting effects on medical research and our understanding of human biology, which could not have been understood without the information from TOHSA.

## Data Availability

The data described in this manuscript are available from https://human-glycome-atlas.org/en/ which will be further updated as the project proceeds.
